# *LAPped in Proof:* LC3‐Associated Phagocytosis and the Arms Race Against Bacterial Pathogens

**DOI:** 10.3389/fcimb.2021.809121

**Published:** 2022-01-03

**Authors:** Bart J. M. Grijmans, Sander B. van der Kooij, Monica Varela, Annemarie H. Meijer

**Affiliations:** Institute of Biology Leiden, Leiden University, Leiden, Netherlands

**Keywords:** LC3-associated phagocytosis, macrophages, neutrophils, autophagy, innate immunity, intracellular pathogens, virulence mechanisms, immune evasion

## Abstract

Cells of the innate immune system continuously patrol the extracellular environment for potential microbial threats that are to be neutralized by phagocytosis and delivery to lysosomes. In addition, phagocytes employ autophagy as an innate immune mechanism against pathogens that succeed to escape the phagolysosomal pathway and invade the cytosol. In recent years, LC3-associated phagocytosis (LAP) has emerged as an intermediate between phagocytosis and autophagy. During LAP, phagocytes target extracellular microbes while using parts of the autophagic machinery to label the cargo-containing phagosomes for lysosomal degradation. LAP contributes greatly to host immunity against a multitude of bacterial pathogens. In the pursuit of survival, bacteria have developed elaborate strategies to disarm or circumvent the LAP process. In this review, we will outline the nature of the LAP mechanism and discuss recent insights into its interplay with bacterial pathogens.

## Introduction

Throughout evolution, microbial pathogens and animal immune cells have developed elaborate mechanisms to face and withstand each other. Understanding these mechanisms lies at the heart of improving medical interventions against microbial infections. Phagocytes, specialized cells of the innate immune system, are characterized by their ability to engulf and intracellularly destroy foreign particles and dying cells. Engulfment and subsequent degradation of microbes is key to our innate, and ultimately adaptive defenses. Central to the phagocytic elimination of microbial invaders is the fusion of the phagosome with lysosomes, a process called phagosome maturation. Unless inhibited by virulence factors, an engulfed microbe will be exposed to an array of lysosomal enzymes — killing it within minutes (Fountain et al., 2021) .

Three different vesicle trafficking mechanisms are known to direct microbial pathogens to lysosomal degradation: phagocytosis, autophagy and LC3‐associated phagocytosis (LAP). All have overlapping characteristics but are initiated *via* distinct pathways, where cargo‐containing vesicles form and mature by different mechanisms. Phagocytosis, which was recognized as early as the 19th century, targets extracellular microbes *via* receptor‐mediated recognition (Rosales and Uribe‐Querol, 2017). Several pathogens have evolved strategies to subvert the phagocytic process, allowing them to establish a niche for their own proliferation (Flannagan et al., 2009). Some microbes, like *Streptococcus pyogenes*, can arrest ingestion by producing toxins or expressing antiphagocytic surface proteins (Brouwer et al., 2016), while others, like *Mycobacterium tuberculosis*, interfere with phagosome integrity to first establish an intravesicular replicative niche and subsequently escape the confines of the phagosomal vesicle (Simeone et al., 2021). Other microbes still, like *Listeria monocytogenes*, take advantage of the acidification of the phagosome, utilizing it to activate virulence-mediated disruption of the phagosomal membrane, leading to immediate invasion of the cytosol (Matereke and Okoh, 2020).

A second way for cells to effectively degrade microbial invaders is autophagy, strictly speaking macroautophagy. By definition, autophagy targets intracellular structures, such as protein aggregates or cytosolic bacteria, capturing them in a characteristic double‐membrane vesicle (Boya et al., 2013). Mediated by a group of conserved autophagy‐related proteins, a cup‐shaped double‐membrane complex is nucleated around a target structure. It extends to seal the target into a closed vesicle, the autophagosome. Similar to phagosomes, the transient autophagosomes mature by fusing with lysosomes. In recent decades, accumulating evidence has illustrated the extensive interactions between microbial pathogens and the host autophagic response, referred to as xenophagy (Deretic and Levine, 2009; Huang and Brumell, 2014). Intracellular pathogens have developed ingenious evasive mechanisms to avoid being killed in the autophagosome. Such strategies include the interference with autophagy‐initiating signaling, disruption of lysosome function and proteolytical inactivation of the autophagic machinery (Jiao and Sun, 2019). Furthermore, microbes have even evolved means to turn host autophagy to their own advantage, utilizing it to foster their own nutrient supply, replication, cellular egress and virulence (Kimmey and Stallings, 2016).

Since 2007, it has become clear that phagocytes have a third degradation mechanism to their disposal, which is now commonly referred to as LAP (Sanjuan et al., 2007). LAP has been described to exist at the crossroads of autophagy and phagocytosis, combining the strengths of both processes to ensure enhanced degradation of the engulfed cargo (Martinez, 2018; Heckmann and Green, 2019). Given its intricate role in anti-microbial immunity and preservation of homeostasis, LAP has sparked much interest in recent years. During LAP, which is initiated by receptor signaling, select parts of the autophagic machinery – particularly the ubiquitin-like protein LC3 (microtubule-associated proteins 1 A/1B light chain) – are specifically recruited to the single-membrane phagosome (Sanjuan et al., 2007). Early in the maturation process, an NADPH oxidase complex is assembled that generates reactive oxygen species (ROS) within the vesicle. Soon after LC3 is conjugated onto the phagosomal membrane, the phagosome (now termed LAPosome) fuses with lysosomes, leading to rapid clearance of the internalized material. LAP is often referred to as a form of non-canonical autophagy, but strictly speaking the term autophagy applies only in relation to the vesicular uptake of cytoplasmic cargo, while LAP targets vesicles with material coming directly from the extracellular environment.

Multiple lines of evidence have demonstrated that LAP mediates a variety of immunological functions that go beyond the elimination of pathogens. The process has been deemed important for the immunotolerant processing of dying cells, regulation of inflammatory responses, establishment of signaling compartments, and even attenuating autoimmunity (Martinez et al., 2015; Heckmann et al., 2017; Wong et al., 2021). With regard to human disease, LAP has drawn particular attention for its role in immunity to different classes of microbial pathogens (Chamilos et al., 2016; Besteiro, 2019; Jiao and Sun, 2019; Akoumianaki et al., 2021). In this review, we focus on the role of LAP in bacterial infectious diseases. We discuss the molecular mechanisms that orchestrate LAP, and provide an overview of its significance in fighting bacterial infections as well as its fragility in view of pathogenic evasion.,

## Mechanisms of LAP Induction and Maturation

While classical autophagy and LAP have significant overlap in their utilization of the molecular machinery, induction of these processes is fundamentally distinct. LAP and related single membrane LC3 lipidation processes are triggered by the engagement of various surface receptors (Sanjuan et al., 2007; Martinez et al., 2015), including Toll-like receptors (TLRs), Dectin‐1, Dectin‐2, but also immunoglobulin receptors such as Fc*γ*R and scavenger receptors such as TIM4 (Sanjuan et al., 2007; Huang et al., 2009; Martinez et al., 2011; Ma et al., 2014; Lamprinaki et al., 2017). In addition, activation of the cytosolic innate immune sensor STING induces LC3 lipidation of single-membrane vesicles (Fischer et al., 2020). How these different cargo engagements and consequent signaling pathways activate the machinery required for LAP remains unclear. However, it has been well documented that LAP proceeds independently of the pre-initiation complex containing ULK1, ATG13, ATG101 and FIP200, which is crucial for autophagy induction (Martinez et al., 2011; Heckmann and Green, 2019). Indeed, LAP typically appears unresponsive to nutrient starvation and other autophagic signals associated with ULK1 activation (Sanjuan et al., 2007). Similar to phagocytosis but unlike classical autophagy, pathogens targeted by LAP are engulfed in a single‐membrane phagosome (Schille et al., 2018). This is one of the most significant ultrastructural differences that distinguishes LAPosomes from classical autophagosomes (Lai and Devenish, 2012).

**Figure 1:**
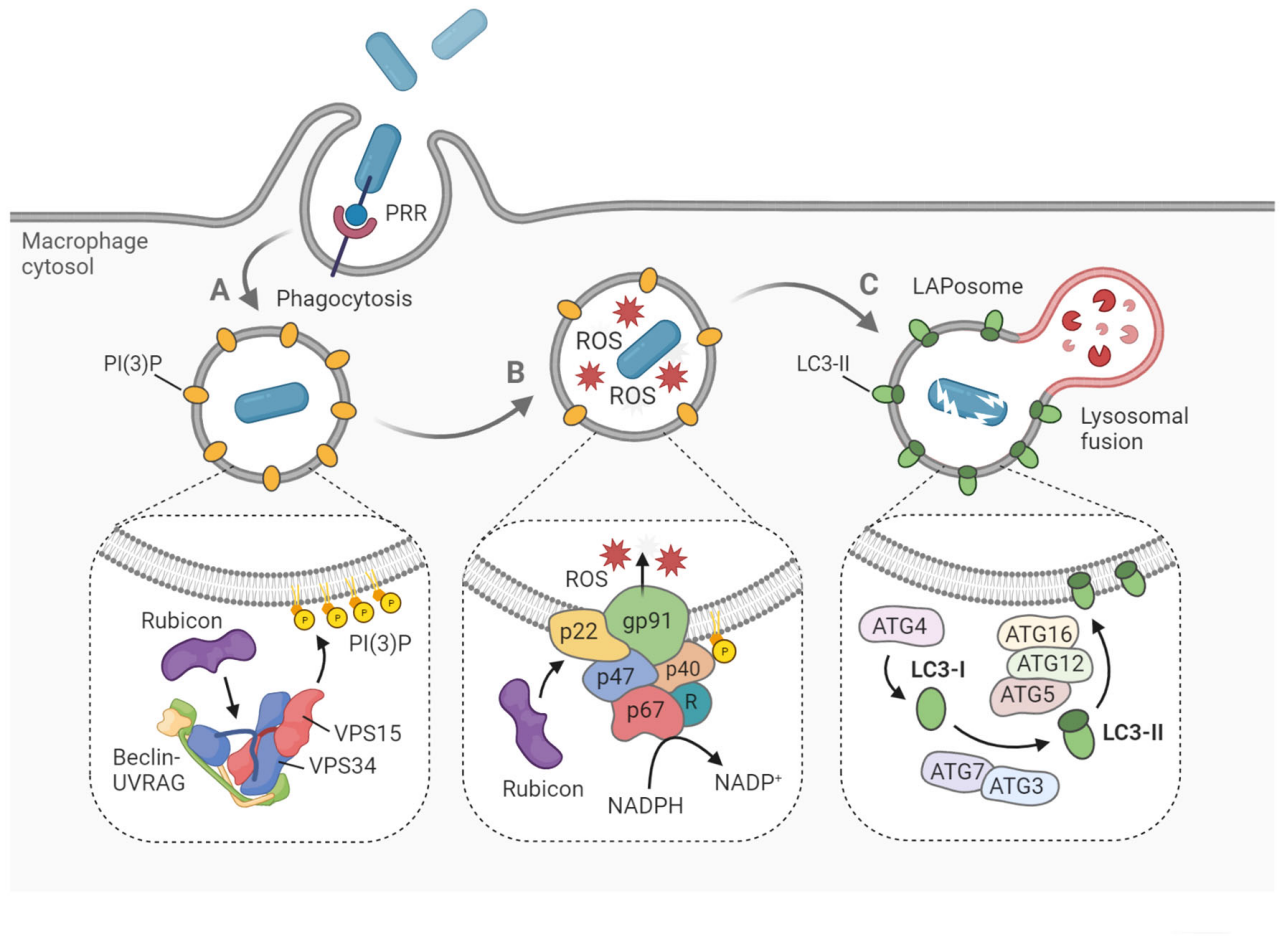
Hallmarks of LAP on the molecular level. LAP begins with pattern recognition receptor (PRR)-mediated phagocytosis of pathogens, dying cells and other particles. **(A)** The phagosome is marked with PI(3)P, a signaling lipid which is generated by the PI3KC3 complex, consisting of Beclin-1, UVRAG, VPS15, VPS34 and Rubicon. **(B)** Within the phagosome, ROS are produced by the NADPH oxidase complex. Rubicon stabilizes the complex *via* interaction with p22^phox^, while p40^phox^ interacts with PI(3)P to recruit the remaining components. **(C)** Cytosolic LC3 is lipidated by the conjugation machinery to form LC3-II on the phagosomal membrane. Soon after, the LAPosome fuses with an available lysosome resulting in rapid degradation of the engulfed cargo. Figure created with BioRender.com.

After the pathogen is internalized, one of the first signaling complexes to associate with the budding phagosome is the class III phosphatidylinositol 3-kinase complex (PI3KC3), which ultimately delivers PI(3)P onto the phagosomal membrane (Matsunaga et al., 2009) (**Figure 1A**). The functional core of PI3KC3 is composed of VPS34 (the catalytic subunit), VPS15 and Beclin-1 (Backer, 2016). Following activation by VPS15 and Beclin-1, VPS34 generates PI(3)P from PI(3) *via* its kinase activity (Volinia et al., 1995; Petiot et al., 2000). The newly formed PI(3)P molecules disseminate throughout the phagosomal membrane, acting as a label for future LC3-conjugation (Martinez et al., 2011). Two critical proteins that specifically recruit PI3KC3 to the phagosome during LAP, are UVRAG and Rubicon (Martinez et al., 2015). While the PI3KC3 complex itself is non-specific for LAP, and is also involved in the activation of classical autophagy, Rubicon is essential for LAP maturation in contrast to its inhibitory role in autophagosome maturation (Martinez et al., 2015). In fact, Rubicon participates at multiple signaling steps relevant for LAP development through interaction with different binding partners (Matsunaga et al., 2009; Yang et al., 2012).

Another hallmark of LAP, which also depends strictly on Rubicon activity, is the generation of reactive oxygen species (ROS) within the phagosome lumen (Martinez et al., 2015) (**Figure 1B**). ROS are produced by the NADPH oxidase 2 complex (NOX2), the only NADPH oxidase complex expressed in phagocytes (Bedard and Krause, 2007). The activity of NOX2 is dependent on the recruitment of four cytosolic subunits, namely p67^phox^, p47^phox^, p40^phox^ and Rac1, to the two membrane-embedded subunits p22^phox^ and gp91^phox^, which form the catalytic center. The p40 protein is capable of direct interaction with the PI(3)P present on the phagosome, functioning as a docking site for the other cytosolic subunits (Ellson et al., 2006). Rubicon is able to stabilize the NOX2 complex *via* direct interaction with p22, resulting in maximal ROS production (Yang et al., 2012). The ROS may serve several roles in the LAP process. Aside from their putative oxidative activity against the pathogen (Slauch, 2011), ROS are needed for recruitment of downstream LAP components, such as ATG7 and LC3 (Lam et al., 2013; Martinez et al., 2015). Furthermore, ROS generation by NOX2 has been shown to cause oxidative inactivation of ATG4B, thereby inhibiting the proteolytic release of LC3 and thus stabilizing the LAPosome (Ligeon et al., 2021). Further aspects of the mechanistic interplay between ROS signaling and LAP maturation are incompletely understood, though it is hypothesized that lipid peroxidation within the phagosome could serve a regulatory function (Holmström and Finkel, 2014).

After the phagosomal membrane is marked by PI(3)P and ROS have been produced, two conjugation systems are activated that will mediate the processing and incorporation of LC3 onto the phagosomal membrane (**Figure 1C**). Cytosolic pro-LC3 is converted into LC3-I by ATG4. Then, LC3-I is lipidated by ATG7-ATG3 and ATG12-ATG5-ATG16L1 *via* covalent attachment on phagosomal surface to form LC3-II (Martinez et al., 2015; Schille et al., 2018). Both LAP and classical autophagy are characterized by the association of LC3-II onto the target membrane. However, recruitment proceeds differently in both processes, as the target membrane is the phagophore in the case of autophagy, and the phagosome in the case of LAP (Herb et al., 2020). Furthermore, autophagy can maintain tissue homeostasis independent of LAP, which has been illustrated by the differential role of ATG16L1 in both processes. Specifically, autophagy requires the ATG5-binding and coiled coil domains of ATG16L1 but not the WD domain, whereas the WD domain is indispensable for LAP (Rai et al., 2019; Fischer et al., 2020; Wang et al., 2021). Following LC3-decoration, the LAPosome will rapidly fuse with lysosomes and acidify (Martinez et al., 2011). While it has been argued that LC3 family proteins play an important role in facilitating this lysosomal fusion, details about the vesicle fusion mechanism remain obscure (Martinez et al., 2015; McEwan et al., 2015; Nguyen and Yates, 2021).

## Biological Functions of LAP

The primary function of LAP is to facilitate the fusion of phagosomes with lysosomes, assuring rapid degradation of the engulfed cargo and regulation of the appropriate immune response (Martinez, 2018). LAP and related single membrane LC3 lipidation processes exhibit a surprising antimicrobial versatility as it is required for successful processing of a wide variety of pathogens across different kingdoms, with the fungal pathogen *Aspergillus fumigatus*, the bacterial pathogen *Listeria monocytogenes*, the parasite *Toxoplasma gondii*, and Influenza A virus as notable examples (Martinez, 2018; Schille et al., 2018; Besteiro, 2019; Herb et al., 2020; Wang et al., 2021). Consequences of aberrant LAP for human disease is now an active field of research (Martinez, 2018; Upadhyay and Philips, 2019). In recent decades, interest in uncovering novel antimicrobial strategies has grown steadily, mainly due to the alarming prevalence of antibiotic resistance leading to incurable bacterial infections (Aslam et al., 2018).

In addition to its antimicrobial functions, LAP has been shown to be relevant for many other immunological processes, such as the clearance of dying cells and apoptotic remnants – a process known as efferocytosis. LAP enables professional phagocytes to process cellular debris in a remarkable immunosilent manner, by keeping levels of pro-inflammatory cytokines and associated signaling pathways at bay (Heckmann et al., 2017). Indeed, Rubicon-deficient mice show a defective clearance of apoptotic cells, resulting in an exaggerated inflammatory phenotype and ultimately the formation of auto-antibodies (Martinez et al., 2016). For humans, proper processing of cellular debris has been shown to be crucial for averting autoimmune disorders, such as systemic lupus erythematosus (SLE) (Muñoz et al., 2010). Intriguingly, genome-wide association studies among SLE patients have found a polymorphism in the ATG5 protein, suggesting that LAP or autophagy might play a critical role in the development of this disorder (Harley et al., 2008; Gateva et al., 2009). Furthermore, defects in LAP have been linked to numerous other inflammatory abnormalities, including atherosclerosis, visceral adiposity, and insulin resistance (Heckmann and Green, 2019).

Contrary to intuition, LAP may be a contributing factor in tumorigenesis, as it has been implicated in the establishment of a conducive microenvironment for cancerous cells. In mice, an increased LAP activity has been associated with tumor growth and aggressiveness (Asare et al., 2020). Indeed, high expression of Rubicon in cancer tissues predicts an adverse survival rate of patients with various cancer types. It is thought that the immunosuppressive signaling networks associated with LAP could be hijacked by developing cancer cells to bypass the immune response, thereby promoting their progression and metastatic potential. The implications of this have been reviewed elsewhere (Asare et al., 2020).

## Interactions of Bacterial Pathogens With LAP

**Figure 2:**
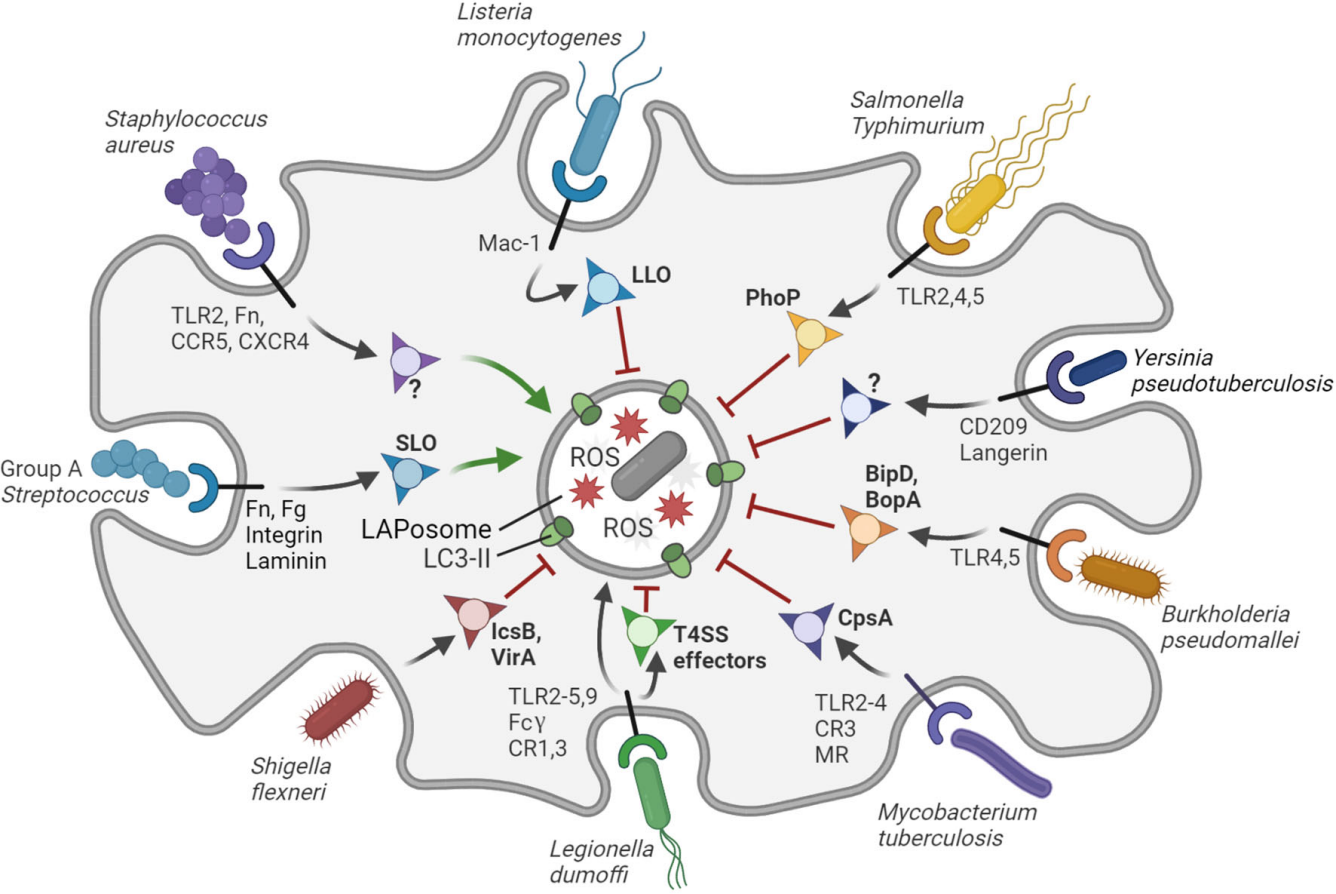
Interactions of bacterial pathogens with LAP. The LAPosome is a single membrane vesicle marked by LC3-II and producing ROS. LAP contributes to host defense, but bacterial pathogens have evolved diverse ways to inhibit (red arrows) or promote (green arrows) LAP to their own benefit. *S. aureus* promotes the formation of LAPosomes in neutrophils *via* an unknown virulence factor to establish a replicative niche. Group A Streptococcus promotes LAP *via* the virulence factor SLO to evade bactericidal xenophagy. *L. dumoffi* is effectively degraded in the LAPosome (black arrow), although it may inhibit LAP to some extent *via* T4SS effector proteins. All other bacterial pathogens shown in the figure can partially inhibit LAP in phagocytes or LAP-like processes in epithelial cells through the virulence factors indicated. Virulence factors that remain to be identified are indicated with question marks. The receptors that mediate entry and/or immune recognition by the host phagocytic cells are shown, except for *S. flexneri*, which attaches to surface proteins of M-cells in the gut epithelium. Figure created with BioRender.com.

The diversity of evasive strategies adopted by different species of pathogens is testament to the complexity and effectiveness of the LAP process. In many cases, evasion mechanisms are only beginning to be discerned on the molecular level. Some bacterial pathogens circumvent LAP altogether by expressing effectors that impair their targeting, while others orchestrate their own internalization and survive inside phagosome (**Figure 2**). Below, we describe some notable examples of LAP-targeted bacterial pathogens and discuss how LAPosome formation and maturation may be modulated by virulence mechanisms of these pathogens (**Table 1**). We have included also cases that may represent different forms of single membrane LC3 lipidation closely resembling LAP.

**Table 1:** Overview of LAP-targeted bacterial pathogens and their evasion strategies.

Pathogen	Mode of entry/innate immune recognition	Virulence factor	Evasion or exploitation of LAP or LAP-like processes	References
*Mycobacterium tuberculosis*	TLR2, TLR4, mannose receptor, complement receptor 3	CpsA, PDIM	Inhibits recruitment of NAPDH oxidase to phagosome. Conceal TLR ligands triggering LAP	Stamm et al. (2015); Köster et al. (2017)
*Listeria monocytogenes*	Mac-1	LLO	Upregulates mitochondrial calcium signaling to acetylate Rubicon	Gluschko et al. (2018); Li et al. (2021)
*Salmonella enterica* serovar t*yphimurium*	TLR2, TLR4, TLR5	PhoP, FlhD, SsrB	Inhibition of phagolysosomal fusion (PhoP), triggering TLR5 and LAP (FlhD), displaying Rubicon-independent virulence (SsrB)	Masud et al. (2019b)
*Legionella dumoffi*	TLR2,3,4,5,9, Fcγ, complement receptor 1,3	Possibly T4SS	Unknown	Hubber et al. (2017)
*Burkholderia pseudomallei*	TLR4, TLR5	BopA, BipD (T3SS efectors)	Escape from LAPosome *via* T3SS	Gong et al. (2011)
*Yersinia pseudotuberculosis*	C-type lectins: Langerin, CD209	Unknown	Interference with LC3. Recruitment through VAMP3, VAMP7	Ligeon et al. (2014)
*Shigella flexneri*	Surface proteins of M-cells	IcsB, VirA, IpaB, OspC3, IpgD	Inhibition LC3 recruitment	Baxt and Goldberg (2014); Campbell-Valois et al. (2015)
Group A *Streptococcus*	Fibronectin, fibrinogen, integrins, laminins	SLO	Evasion of xenophagy by inducing LAP	Lu et al. (2017)
*Staphylococcus aureus*	TLR2, CCR5, CXCR4, fibronectin	Unknown	Establishing a LAP-dependent replication niche	Prjasnar et al. (2021)

### Mycobacterium tuberculosis

*Mycobacterium tuberculosis* is the causative agent of acute or chronic manifestations of tuberculosis, the most lethal bacterial infectious disease today (WHO, 2020). *M. tuberculosis* is recognized and phagocytosed by macrophages *via* different surface receptors, including TLRs, mannose receptors and complement receptors (Schlesinger, 1993; Yu et al., 2014). Even though *M. tuberculosis* has been shown to be targeted by LAP, it is still not clear which fraction of phagosomes progresses to LAPosomes and whether this process enhances the ability of phagocytes to clear the pathogen or it is exploited by the pathogen for its intracellular survival (Köster et al., 2017; Köster et al., 2018).

LAP resistance of *M. tuberculosis* was found to be mediated by the virulence factor CpsA, which prevents recruitment of NOX2 to the pathogen-containing phagosome (Köster et al., 2017; Köster et al., 2018). While the inactivation of NOX2 by CpsA resulted in impaired lysosomal trafficking, reduced phagolysosome biogenesis and ultimately the survival and proliferation of intracellular *M. tuberculosis*, the deletion of CpsA in *M. tuberculosis* resulted in efficient degradation of the pathogen by LAP. *M. tuberculosis* is known to secrete several virulence factors that interfere with phagosome maturation and thus, it is likely to evade LAP in different ways. In this regard, the virulence factor NdkA has been shown to contribute to intracellular survival by interfering with phagosome maturation (Sun et al., 2010). Moreover, the presence of NdkA has been shown to decrease the recruitment of p67^phox^ and Rac1 to the phagosome, interfering with ROS production by the NADPH oxidase complex and presumably undermining LAP (Sun et al., 2013).

In addition to inhibiting phagosome maturation, *M. tuberculosis* is able to conceal its presence by manipulating TLR recognition and therefore preventing its phagocytosis. It is known that TLR4 recognizes lipids, glycoproteins, secreted proteins and other surface ligands from *M. tuberculosis*, leading to fast phagocytosis of the pathogen (Stamm et al., 2015). Absence of phthiocerol dimycocerosate lipids (PDIM) in the *M. tuberculosis* cell wall induced an increase of TLR-dependent recruitment of microbicidal macrophages, indicating the inhibitory role of PDIM on pathogen recognition (Cambier et al., 2014). It has been suggested that this TLR recognition inhibition is due to masking of the mycobacterial pathogen associated molecular patterns (PAMPs) by PDIM (Cambier et al., 2014). The study of PDIM adds another dimension by which evasion of the LAP mechanism by *M. tuberculosis* is possible.

A screen in planarian flatworms identified a protein MORN2, of which the human ortholog was shown to play an important role in the LAP response of macrophages to *M. tuberculosis*, and also *L. pneumophila* and S. aureus (Abnave et al., 2014). MORN2 promotes LC3 recruitment to *M. tuberculosis*-containing phagosomes and their maturation into phagolysosomes. The role of LAP was confirmed by demonstrating the single-membrane nature of the bacteria-containing vesicles as well as the requirement of Atg5 and Beclin1 for LC3 recruitment, but not Ulk1 and Atg13 (Abnave et al., 2014). Further in line with the proposed role of MORN2 in LAP, its function was shown to depend on ROS (Morita et al., 2020). Using *Escherichia coli* and zymosan as alternative LAP substrates, SNARE proteins like SNAP-23 and syntaxin11 were implicated in vesicle fusions during MORN2-mediated LAP (Morita et al., 2020). Altogether, MORN2 emerges from this work as a positive regulator of LAP, which warrants further studies with *M. tuberculosis* and other pathogens.

In conclusion, while there is evidence that *M. tuberculosis* is actively targeted by LAP, its modes of evasion are still starting to be understood. Evasion of LAP by *M. tuberculosis* is likely to occur during the maturation of the phagosome while, among others virulence factors, CpsA and NdkA are secreted. Considering the diversity of virulence factors known to affect phagosome maturation, it is expected that *M. tuberculosis* mutant screens will soon reveal additional effectors critical for phagosome maturation and anti-LAP virulence. Additionally, evasion of LAP initiation *via* phagocytosis has been observed, a process mediated by effector molecules such as PDIM cell wall lipids. MORN2 seems a useful addition to Rubicon for further study as a host factor specifically promoting LAP.

### Listeria monocytogenes

*Listeria monocytogenes* is an opportunistic bacterium that can cause severe food-borne diseases in immunocompromised individuals, pregnant women and newborns (Cossart and Lecuit, 1998; Lam et al., 2013). Clearance of *L. monocytogenes* is explicitly promoted by LAP and this host-pathogen interaction can be regarded as one of the most striking examples of the microbicidal power of the LAP pathway (Gluschko et al., 2018; Herb et al., 2018).

During infection, *L. monocytogenes* utilizes the virulence factors listeriolysin (LLO) and PlcA/B to escape from the phagosome and enter the cytosol, where it acquires actin-based motility (Cossart and Lecuit, 1998; Seveau, 2014). Within the cytosol, *L. monocytogenes* actively inhibits classical autophagy *via* IcsB, ActA and PlcA/B (Birmingham et al., 2008; Lam et al., 2013). LAP has become known as the key mechanism providing anti-Listeria immunity (Gluschko et al., 2018). Recognition of *L. monocytogenes via* the β2 integrin Mac-1 receptor activates LAP and the associated phagosomal ROS response. Interestingly, the same study also provided evidence that the execution of LAP is not influenced by virulence factors that inhibit classical autophagy and revealed a crucial role for acid sphingomyelinase, broadening our understanding of the LAP mechanism. The acid sphingmyelinase protein facilitates alterations in the lipid composition of the membrane, allowing the subsequent activation of the Nox2 complex, crucial for the ROS production and subsequent LC3 recruitment (Gluschko et al., 2018).

The possible evasion of LAP by *L. monocytogenes* is only beginning to be understood. Recent work showed that *L. monocytogenes* is able to suppress LAP by modulating mitochondrial calcium signaling (Li et al., 2021). After phagocytosis, *L. monocytogenes* induces mitochondrial calcium uptake by the mitochondrial Ca^2+^ uniporter (MCU) transporter. This increased calcium uptake promotes the production of acetyl-coenzyme A (acetyl-CoA) by pyruvate hydrogenase. Outside the mitochondrion, Rubicon is acetylated by acetyl-CoA resulting in decreased activity of Rubicon in the LAP pathway, thus acting in favour of bacterial survival. In agreement, a knockout of the MCU transporter abolishes calcium uptake, allowing LAP to overpower *L. monocytogenes* infection (Li et al., 2021). Together, these results show that *L. monocytogenes* is able to inhibit LAP through eliciting mitochondrial signaling, which adds to the growing connective network between mitochondrial metabolism and innate immune defense mechanisms (Li et al., 2021).

Interestingly, there are also cases known in which *L. monocytogenes* induces a prolonged infection which LAP fails to control. This might be achieved due to the formation of certain compartments termed spacious Listeria-containing phagosomes (SLAPs) *via* manipulation of the LAP mechanism. It is believed that these single membrane compartments provide a niche in which the bacteria are able to replicate and proliferate. The formation of this niche is possible due to failure of LAP to clear the infection and a lack of the expression of virulence factors that mediate escape from phagosomes into the cytosol (Birmingham et al., 2008; Lam et al., 2013). However, it should be noted that SLAPs were observed in immunodeficient or oncogenic transformed cells, and therefore their formation in healthy macrophages remains unclear.

Recent studies revealed that phagosome permeabilization by *L. monocytogenes* triggers another single membrane LC3 lipidation pathway, which has been named pore-forming toxin-induced non-canonical autophagy pathway (PINCA) (Mitchell et al., 2018; Gluschko et al., 2021). In bone marrow-derived macrophages, *L. monocytogenes* was shown to be targeted sequentially by multiple autophagic processes. The LLO-mediated perforation of phagosomes was shown to trigger LC3 recruitment in an ULK1-independent process. However, this PINCA response had no role in restricting bacteria growth, in contrast to subsequent xenophagy, which defends against *L. monocytogenes* bacteria upon invasion of the cytosol (Mitchell et al., 2018). PINCA is distinct from LAP, because it can occur in NOX2-deficient macrophages (Gluschko et al., 2021). Induction of LAP in PINCA-competent cells confirmed that LAP contributes to host defense, while no clear anti-*Listeria* function of PINCA could be identified (Gluschko et al., 2021).

To summarize, LAP provides anti-*Listeria* immunity in macrophages, while PINCA, the LC3 recruitment to permeabilized phagosomes, does not restrict bacterial growth (Gluschko et al., 2018; Herb et al., 2018; Mitchell et al., 2018; Gluschko et al., 2021). The host defense function of LAP is counteracted by bacterial LLO, the primary virulence factor that mediates invasion of the cytosol after phagocytosis, where *L. monocytogenes* has to defend itself against xenophagy (Seveau, 2014; Osborne and Brumell, 2017; Mitchell et al., 2018). In addition, it was recently reported that *L. monocytogenes* is able to suppress LAP by manipulating the MCU transporter and redirecting calcium signaling to inhibit the key LAP host factor, Rubicon (Li et al., 2021). Many cases of prolonged infections of *L. monocytogenes* are known, suggesting that bacterial virulence mechanism can modulate LAP to clear the way for SLAP biogenesis (Lam et al., 2013). The distinctive roles of LAP and PINCA and the mechanistic differences between these two processes require further dissection. It will be of great interest to investigate how these two mechanisms may also function side by side in infections with other pathogens that permeabilize phagosomes.

### Salmonella typhimurium

*Salmonella enterica* serovar Typhimurium (*S. typhimurium*) is an intracellular pathogen that can invade both non-myeloid and phagocytic cells and is a major cause of gastroenteritis (Ibarra and Steele-Mortimer, 2009). Early studies on Salmonella infection in mouse macrophages and human epithelial cells already suggested that LAP could be a critical player in the immune response, because the triggering of TLR or Fc-gamma receptors induced LC3 recruitment on phagosomes in a manner dependent on ROS production (Huang et al., 2009). The requirement of phagocytic NADPH oxidase for LC3 recruitment to macrophage phagosomes was confirmed by knockdown of the Cyba component of NOX2 in a zebrafish embryo model of systemic *S. typhimurium* infection (Masud et al., 2019a). Furthermore, knockdown of Atg5 and Rubicon, but not the autophagy preinitiation factor Atg13, were shown to be required for LC3 recruitment and for the successful clearance of bacteria in the zebrafish model, providing *in vivo* evidence for the anti-Salmonella function of LAP (Masud et al., 2019a).

While LAP provides protection to *S. typhimurium* infection in zebrafish embryos, there is still a high mortality rate, indicating that the pathogen can resist LAP to a certain extent (Masud et al., 2019a). Several mutant *S. typhimurium* strains were screened to determine the possible role of virulence factors in LAP evasion (Masud et al., 2019b). None of the virulence factors tested, PhoP, PurA, FlhD, SipB and SsrB, appeared to be necessary for the host LAP response, as mutations in these factors did not abolish Rubicon-dependent GFP-LC3 recruitment (Masud et al., 2019b). However, quantitative differences in GFP-LC3 recruitment were observed between the wild type and mutant strains. The PhoP and PurA deficient strains, both attenuated in zebrafish and other animal models, respectively elicited higher and lower GFP-LC3 recruiment (Masud et al., 2019b; Garvis et al., 2001; Thompson et al., 2011; Dalebroux and Miller, 2014). The PhoP regulon has been reported to reduce TLR activation, serve a role in the inhibition of the phagolysosomal fusion, and mediate adaption to intra-macrophage stress (Garvis et al., 2001; Thompson et al., 2011; Dalebroux and Miller, 2014). Therefore, the higher levels of GFP-LC3 recruitment in infection with the Δ*phoP* mutant could suggest a role for PhoP in LAP evasion (Masud et al., 2019b). In contrast, in the case of Δ*PurA* mutant bacteria, a strongly reduced GFP-LC3 recruitment was observed, which might be explained by the virtually complete loss of virulence of this mutant, which could lead to rapid clearance of most of the bacterial population without inducing signals for LAP. (O’Callaghan et al., 1988; Masud et al., 2019b).

Mutation in the FlhD gene, which is crucial for flagella formation of *S. typhimurium*, strongly reduced GFP-LC3 recruitment in the zebrafish model. In line with results in mice, FlhD mutation also resulted in hypervirulence of the *S. typhimurium* pathogen in zebrafish (Fournier et al., 2009). An explanation for both the reduced GFP-LC3 recruitment and the hypervirulence could be that LAP induction is dependent on the recognition of flagellin by TLR5. However, to date no direct link between the signaling of TLR5 and LAP has been established, and therefore the role of the TLR ligand receptor interaction in LAP remains to be studied.

Finally, all the above-mentioned *S. typhimurium* strains displayed increased virulence in a Rubicon-deficient zebrafish host, with the notable exception of a Δ*SsrB* mutant (Masud et al., 2019b). SsrB is part of the bacterial regulatory system controlling expression of *Salmonella* Pathogenicity Island 2 (SPI2) effector molecules that are required for maintenance of the *Salmonella*-containing vacuole (Walthers et al., 2007). Knockdown of zebrafish Rubicon led to reduced GFP-LC3 recruitment towards Δ*SsrB* mutant bacteria, similar as observed with wild type bacteria or other virulence mutants. However, ΔSsrB survival was unaffected by Rubicon knockdown, suggesting that SPI2 effectors could be important for intracellular replication of *S. typhimurium* under conditions where LAP is impaired (Masud et al., 2019b).

To sum up, *S. typhimurium* is a pathogen which is targeted by the LAP pathway that is crucial for proper pathogen clearance, possibly triggered by TLR5-mediated recognition of flagella. Although successful engulfment and degradation is observed, it is possible that virulence factors like the PhoP/Q operon contribute to LAP evasion. Unlike other wild type or mutant Salmonella strains, Δ*SsrB* mutants, impaired in the expression of SPI2 effectors, were unable to display increased virulence in a LAP-deficient zebrafish host. The specific SPI2 effector(s) responsible for this phenotype remain to be established.

### Legionella dumoffii

*Legionella dumoffii* is an intracellular pathogen which can reside in the vacuole after phagocytosis, and is closely related to the human lung disease pathogen, *Legionella pneumophila* (Horwitz, 1983). Phagocytosis of *Legionella* species is mediated by the CR1 and CR3 complement receptors and the Fcy receptor, and innate immune recognition of cell wall components, flagella and bacterial DNA is facilitated by among others the TLR2,3,4,5,9 receptors (Husmann and Johnson, 1992; Grigoryeva and Cianciotto, 2021). Upon phagocytosis, a subpopulation of *L. dumoffii*-containing single-membrane vesicles is decorated with LC3, which requires Rubicon and NOX2 activity, indicating that maturation of these vesicles occurs *via* the LAP pathway (Hubber et al., 2017). In addition, the initiation of the LAP response towards L. dumoffii requires pathogen recognition *via* TLR2 and diacylglycerol signaling. There was no interaction of *L. dumoffii* with ubiquitin receptors and LC3 decoration was independent of ULK1 kinase, thus arguing against a role for selective autophagy and supporting that a subpopulation of *L. dumoffii* resides in LAPosomes (Hubber et al., 2017).

Interestingly, the formation of *L. dumoffii*-containing LAPosomes is dependent on the presence of the bacterial type four secretion system (T4SS). However, independent of LAP, the majority of the bacteria-containing phagosomes are remodelled into a compartment that resembles the endoplasmatic reticulum, thereby inhibiting the fusion with lysosomes and allowing replication (Hubber et al., 2017). Similar to LAP this process is also mediated *via* the activity of T4SS, but it is not understood what determines if expression of T4SS leads to evasion of the immune system or directs bacteria to LAP-mediated degradation. To date *L. dumoffii* remains a relatively poorly studied pathogen compared to other pathogens. Further research involving *L. dumoffii* should be performed to create a more in depth understanding of the interaction between LAP and *L. dumoffi*.

### Burkholderia pseudomallei

*Burkholderia pseudomallei* is a soil-dwelling pathogen that causes pneumonia, skin changes and sometimes severe inflammatory cascades and lethal sepsis, a condition known as melioidosis (Wiersinga et al., 2007). It is phagocytosed by macrophages, neutrophils and dendritic cells, and capable of invading epithelial cells (Horton et al., 2012). The pathogen is recognized by TLR2 and TLR4, but TLR2 has been shown to impact negatively on the host defense function, suggesting that this TLR is responsive for severe dysregulation of the immune system and/or facilitates the creation of a bacterial replication niche (Wiersinga et al., 2007). The type III secretion system (T3SS) of *B. pseudomallei* is required for its escape from phagosomes, permitting replication in the cytosol (Cullinane et al., 2008).

*B. pseudomallei* was found to co-localize with LC3 during infection of mouse RAW 264.7 macrophages and resides in single-membrane compartments, characterized as LAPosomes (Cullinane et al., 2008; Gong et al., 2011; Li et al., 2013). Starvation but not rapamycin treatment enhanced the residence *B. pseudomallei* in these LAPsomes, a process requiring Beclin 1 activity (Li et al., 2013). Treatment of RAW264.7 macrophages with lipopolysaccharide (LPS) from *B. pseudomallei* increased GFP-LC3 puncta formation, while removal of LPS decreased this response. Considering that the effect of *B. pseudomallei* LPS is mediated by TLR4 and unexpectedly also TLR2, it was proposed that LPS induces LAP in a TLR-dependent manner during *B. pseudomallei* infection (Wiersinga et al., 2007; Gong et al., 2011). By mediating the escape from phagosomes, the T3SS facilitates evasion of the LAP mechanism (Cullinane et al., 2008; Gong et al., 2011). Mutant bacteria for the bopA and bipD proteins, both crucial for the T3SS, show diminished escape from the phagosome, indicating the importance of a proper functioning T3SS for evasion of LAP (Cullinane et al., 2008; Gong et al., 2011).

The role of T3SS could in theory be exploited to increase the susceptibility of *B. pseudomallei* to LAP. T3SS-associated ATPases are known to be crucial for the proper function of the TTSS3 and therefore represent possible targets for modulating the interaction of the pathogen with LAP. Small-molecule inhibitors for the T3SS ATPase have been identified and are used to study the effect on *B. pseudomallei* infection and LAP. One of the ATPase inhibitors counteracted the escape of bacteria from the phagosome, leading to increased targeting by LAP and reduced bacterial survival. These promising results could be important for the development of therapies aimed against *B. pseudomallei* infections (Gong et al., 2015).

### Yersinia pseudotuberculosis

*Yersinia pseudotuberculosis* is another food-born pathogen capable of causing an enteric illness. It can infect both epithelial cells and phagocytes by binding to integrins (Isberg and Leong, 1990; Pujol and Bliska, 2003). After invading a phagocyte, *Y. pseudotuberculosis* can survive inside the cell by manipulating the autophagy machinery and impairing the acidification of the autophagosome (Moreau et al., 2010). However, in epithelial cells *Y. pseudotuberculosis* was found to be captured in LC3-decorated, single-membrane and non-acidic vesicles. Despite that epithelial cells are non-phagocytic and lack the NOX2 complex required for LAP, the response of these cells to *Y. pseudotuberculosis* is reminiscent of LAP and could represent a related mechanism (Ligeon et al., 2014).

The study of *Y. pseudotuberculosis* in epithelial cells focused on the role of host derived, vesicle-associated membrane proteins (SNARE proteins) in the LAP-like response (Ligeon et al., 2014). At least two of the SNARE family members, VAMP3 and VAMP7, were found to be involved in the recruitment of LC3 to the pathogen-containing vesicles. Overexpression of VAMP3 resulted in an increase of *Y. pseudotuberculosis* bacteria localized in single-membrane vesicles. Conversely a knockdown of VAMP3 resulted in an increase of *Y. pseudotuberculosis* bacteria localized into double-membrane vesicles. These results suggest that a high concentration of VAMP3 increases LAP-like activity and a low concentration of VAMP3 increases the activity of the classical autophagy. In other words, VAMP3 appears to function as a molecular checkpoint for commitment to the single membrane pathway (LAP-like) or to the double membrane pathway (classical autophagy), dependent on its expression level. VAMP7 associates with the single membrane vesicles after the recruitment of VAMP3. Knockdown of VAMP7 led to a decrease in LC3 decoration of the single membrane compartments, suggesting that VAMP7 protein mediates LC3 recruitment during the LAP-like process. It should be noted that the VAMP7 protein also participates in the recruitment of LC3 during the classical form of autophagy, thereby suggesting a double role for VAMP7 of which the mechanism still remains unknown (Ligeon et al., 2014).

Evasion of the LAP-like response by *Y. pseudotuberculosis* is presumably mediated by blocking the acidification of the phagosome, something which is also seen in classical autophagy (Ligeon et al., 2014). Both the LAP-like process and autophagy are manipulated to establish a non-acidic niche, which raises the question how the manipulation of these two mechanisms is mediated and which processes contribute to their development. Multiple studies showed that SNARE proteins like VAMP3 and VAMP7 could be key to determine the maturation of different vesicular pathways (Fader et al., 2009; Itakura et al., 2012; Moreau et al., 2013).

Concluding, single membrane LC3 lipidation mechanism similar to LAP seems to target the *Y. pseudotuberculosis* pathogen in epithelial cells, but evasion of this mechanism by inhibition of LAPosome maturation is observed, leading to the formation of a replication niche (Ligeon et al., 2014). The VAMP3 protein seems to be a molecular switch for commitment to the single membrane or double membrane pathways. Additional evidence indicated a role for VAMP7 in LC3 recruitment during the LAP-like response, similar as in classical autophagy (Ligeon et al., 2014). It remains to be established whether or not this response also plays a prominent role in other cell types, including phagocytes.

### Shigella flexneri

*Shigella flexneri* is a pathogen that invades epithelial cells and is targeted by a LAP-like mechanism early during infection, but is capable of effectively evading this host defense reponseby escaping into the cytosol and acquiring actin-based motility similar to *L. monocytogenes* (Baxt and Goldberg, 2014). It has been found that the presence of the T3SS is crucial to induce the uptake of *S. flexneri*, followed by the initiation of the LAP-like process (Campbell-Valois et al., 2015). IcsB and VirA are secreted effector proteins involved in the escape of the pathogen from the LC3-decorated vesicle into the cytosol, and therefore these virulence factors are also crucial for the evasion of the LAP-like pathway. (Baxt and Goldberg, 2014; Campbell-Valois et al., 2015).

Toca-1 is a host-derived protein required for the formation of actin tails that propel *S. flexneri* (Leung et al., 2008). The interaction of Toca-1 with IcsB was found to inhibit LC3 recruitment, presumably by inhibiting the ATG5 protein, which is crucial for the recruitment of LC3 to the phagosome (Baxt and Goldberg, 2014). Recent results also indicated that Toca-1, besides interacting with IcsB, also interacts with several other *S. flexneri* effectors, namely IpaB, OspC3 and IpgD. The function of these interactions and possible role in the evasion of the LAP-like response, autophagy and other aspects of *S. flexneri* pathogenesis remains to be further investigated (Miller et al., 2018).

### Group A *Streptococcus* and *Streptococcus pneumoniae*

Group A *Streptococci* (GAS), mostly belonging to the species *Streptococcus pyogenes*, are commonly found among the bacteria colonizing the throat and skin, but they can also cause a range of mild to severe infections, including the deathly toxic shock syndrome (Henningham et al., 2012). Similarly, *Streptococcus pneumoniae*, which is not classified under GAS, generally colonizes the nasopharynx, but can become a cause of pneumonia, septicemia and meningitis (Bogaert et al., 2004). *Streptococci* adhere to various host cell surface receptors, among which fibronectin, fibrinogen, integrins and laminins (Brouwer et al., 2016). Recent studies have implicated LAP in the innate immune defense against both GAS and *S. pneumoniae* (Lu et al., 2017; Cheng et al., 2019; Inomata et al., 2020; Ogawa et al., 2020; Shizukuishi et al., 2020).

GAS is able to survive and replicate in endothelial cells. While these cells are autophagy competent under starvation, they were unable to sequester GAS in autophagosomes, which could be attributed to defective ubiquitin recruitment (Lu et al., 2017). The endothelial cells did capture GAS inside single membrane, LC3-associated vesicles. However, these GAS-containing vesicles failed to properly acidify after fusion with lysosomes and therefore bacterial clearance was impaired (Lu et al., 2017). NOX2 but not ULK1 was found to colocalize with the LC3-positive GAS-containing vesicles, indicating that they arise by LAP (Cheng et al., 2019). Inhibition of ROS production *via* NOX2, restored the vesicle acidification, redirected LAP to conventional anti-bacterial autophagy, and thereby reduced the intracellular growth of GAS. Furthermore, it was shown that streptolysin O (SLO) induces LAP and associated ROS production *via* β1 integrin. Thus, GAS evades the conventional, bacteriostatic autophagy route and induces a largely ineffective LAP response *via* its virulence factor SLO.

In the case of *S. pneumoniae*, LC3 association was investigated both in non-myeloid cells (fibroblasts) and in macrophages (Inomata et al., 2020; Ogawa et al., 2020; Shizukuishi et al., 2020). In non-myeloid cells it was observed that a LAP-like process and canonical autophagy are deployed sequentially, with the formation of LAPosome-like vesicles being indispensable for subsequent autophagosomes formation (Ogawa et al., 2020; Shizukuishi et al., 2020). In contrast to the LAP pathway, the *S. pneumoniae*-containing vesicles that resemble LAPosomes acquire LC3 independently of ROS. However, a feature shared with LAP is that their formation does not require FIP200, a component of the autophagy preinitiation complex. It was observed that that interactions between SQSTM1/p62 and ATG16L1 PcLV are required for the formation of the LAPosome-like vesicles and that LC3 and NDP52 (a member of the SQSTM1/p62 family) disappeared from these vesicles prior to the transition of the bacteria to autophagomes (Ogawa et al., 2020). What precisely distinguishes this LAP-like process from LAP, and whether the two processes can be operative simultaneously, requires further investigation.

In murine bone marrow-derived macrophages, a common LAP response to *S. pneumoniae* was observed where formation of LC3-positive, single membrane vesicles required Rubicon, NADPH oxidase, Atg5 and Atg7, but none of the autophagy preinitiation factors, Ulk1, FIP200, and Atg14 (Inomata et al., 2020). While highly efficient in macrophages from young mice, this LAP pathway was defective in macrophages from old mice, making them deficient in bacterial killing. Concomitant with the loss of LAP, macrophages from older mice also produced high levels of inflammatory cytokines. These interesting findings suggest that diminishing of LAP with age contributes to inflammation and infection susceptibility (Inomata et al., 2020).

### Staphylococcus aureus

*S. aureus* can cause a wide range of diseases, from local skin infections to fatal bacteremia, often associated with antibiotic resistance (Lowy, 1998). While known for its extensive extracellular growth ability in infected tissues, intracellular growth stages in host phagocytes were recently found to be crucial for *S. aureus* pathogenicity (Prajsnar et al., 2012; McVicker et al., 2014). The internalization of *S.aureus* and its recognition is mediated by several surface proteins and receptors, including fibronectin, TLR2, and chemokine receptors like CCR5 and CXCR4 (Edwards et al., 2010; Bi et al., 2015; Tam et al., 2016).

Studies into the autophagy response to *S. aureus* led to different outcomes, pointing either to a host-beneficial effect or suggesting that the pathogen takes advantage of the host autophagy machinery (reviewed in Munoz-Sanchez et al., 2020). Similarly, the host LAP pathway has been found to be exploited to the pathogen’s benefit (Prajsnar et al., 2021). In a zebrafish systemic infection model, *S. aureus* was found to establish an intracellular niche in neutrophils. When internalized by these phagocytes, *S. aureus* was rapidly decorated by GFP-LC3, forming spacious GFP-LC3-positive vacuoles that did not acidify. Chemical and genetic disruption of NADPH oxidase prevented GFP-LC3 recruitment, indicating that the replication niche is formed by LAP, although the role of Rubicon was not addressed. Autophagy played an antagonistic role in this infection model, as GFP-Sqstm1 (p62) also decorated a subset of bacteria and Sqstm1 knockdown impaired host survival. Thus, despite a protective effect of selective autophagy, the prevailing LAP response in zebrafish neutrophils contributes to *S. aureus* pathogenesis and inhibition of this response improves host resistance (Prajsnar et al., 2021). The *S. aureus* virulence factors involved in generating the spacious LAPosomes and preventing acidification are yet to be uncovered.

## Conclusions and Perspectives

When the LAP process was first described, its importance for microbial control was already underlined (Sanjuan et al., 2007). In the years that followed, it became clear that LAP constitutes a critical cornerstone for host defense against a variety of bacterial invaders. We now know that *M. tuberculosis*, *L. monocytogenes*, *L. dumoffi*, *S. Typhimurium*, *B. pseudomallei*, among other pathogens discussed in this review, are captured in a LC3-II-positive single-membrane phagosome and require Rubicon and NOX2-driven ROS production for their clearance. As the list of bacteria targeted by LAP continues to grow, efforts have been dedicated to determine how LAP affects the pathology of infectious disease. LAP has been best characterized in macrophages, yet LC3 lipidation of phagosomes has recently also been demonstrated in neutrophils, albeit as a mechanism of bacterial pathogenesis (Prajsnar et al., 2021). Most of the knowledge on LAP is based on genetic analyses of NOX2 and Rubicon, which also have LAP-independent roles in host defense and autophagy that complicate the interpretation of data. Furthermore, much remains to be discovered about the mechanisms downstream of NOX2 and Rubicon and about mechanisms independent of these two factors, especially because multiple pathways to single membrane LC3 lipidation seem to exist (Mitchell et al., 2018; Rai et al., 2019; Fischer et al., 2020, Gluschko et al., 2021 Wong et al., 2021).

Future research should lead to better understanding of the discrete mechanisms and functions of LAP and LAP-like processes, such as PINCA, which is triggered by phagosome permeabilization rather than by NOX2 activity (Mitchell et al., 2018; Gluschko et al., 2021). Another important area for future research is how LAP might work in concert with the closely related and recently discovered process, LC3-associated endocytosis (LANDO) (Heckmann and Green, 2019). LANDO has been shown to regulate the turnover of Aβ receptors in a murine model of Alzheimer’s disease. It will be of great interest to explore if LANDO and LAP also control levels of pattern recognition receptors and thereby contribute to the regulation of the innate immune response and pathogen clearance.

Although the different evasive strategies that bacteria use to circumvent or take advantage of LAP are progressively being unraveled, many questions about the molecular mechanisms that undergird these strategies remain unexplored. Strikingly, most bacterial pathogens targeted by LAP have evolved ways to specifically interfere with NOX2, signifying the central importance of NOX-derived ROS in LAP maturation. It is still difficult to say if this importance arises from the microbicidal or rather from the signaling functions of ROS (Holmström and Finkel, 2014), although this seems to differ between bacterial species (Herb and Schramm, 2021). Redox regulation of ATG proteins is indeed a prerequisite for the production of LC3-II during autophagy (Scherz-Shouval et al., 2007). Recently, it was discovered that NOX2 has a role in stabilizing the LAPosome itself by safeguarding LC3-II *via* redox regulation of ATG4B (Ligeon et al., 2021). Future studies should seek to answer how ROS contribute to pathogen clearance and engage parts of the LAP machinery, like the LC3 conjugation systems.

The ways in which LAP enhances phagosome-lysosome fusion are incompletely understood. Different bacterial effectors such as *Mycobacterium* CpsA and *Legionella* RavZ have been associated with impaired lysosomal trafficking during LAP (Choy et al., 2012; Köster et al., 2017). Such effectors may be critical for LAP evasion. However, as phagosome–lysosome fusion is a highly dynamic process that depends on membrane lipid composition and the coordinated action of Rab GTPases, tethering factors and SNAREs (Nguyen and Yates, 2021), details of the evasion strategies counteracting lysosomal fusion have yet to be substantiated.

At present, the machinery required for LAP can be specifically manipulated by various pharmacological or genetic means, such as the recently developed Rubicon inhibitor TIPTP (Kim et al., 2020), as well as Rubicon- and ATG16L1-deficient mouse lines (Martinez et al., 2016; Rai et al., 2019). Together, these techniques will be of great use to elucidate how bacterial species are targeted and killed by LAP, leaving aside the confounding effects of classical autophagy. Better knowledge about the antibacterial effects of LAP, and the comparison with antifungal and antiparasitic LAP mechanisms, could provide vital clues for developing novel intervention strategies in the ongoing battle against infectious diseases.

In most circumstances, cells that are proficient in LAP are generally well equipped to combat bacterial infection. However, some pathogens, with *S. aureus* as a notable example, are able to exploit LAP to generate a replication niche. In time, our understanding of LAP and its links with infectious disease will continue to increase in scope and diversity. It is, in the words of Shakespeare, a pathway *lapp’d in proof* – that is, clad in strong (proven) armor – when it comes to virulent bacteria that continue to undermine our vulnerable immune systems.

## Author Contributions

BG and SK performed the literature research, wrote the manuscript, and designed the figures. MV made textual revisions. AM supervised the literature research and made textual revisions. All authors checked and approved the final version.

## Funding

Our research related to this review is funded by the European Union’s Horizon2020 Marie Sklodowska-Curie projects H2020-MSCA-IF-2014-655424 and 721537-ImageInLife.

## Conflict of Interest

The authors declare that the research was conducted in the absence of any commercial or financial relationships that could be construed as a potential conflict of interest.

## Publisher’s Note

All claims expressed in this article are solely those of the authors and do not necessarily represent those of their affiliated organizations, or those of the publisher, the editors and the reviewers. Any product that may be evaluated in this article, or claim that may be made by its manufacturer, is not guaranteed or endorsed by the publisher.
